# Degalactotigonin, a Steroidal Glycoside from* Solanum nigrum*, Induces Apoptosis and Cell Cycle Arrest via Inhibiting the EGFR Signaling Pathways in Pancreatic Cancer Cells

**DOI:** 10.1155/2018/3120972

**Published:** 2018-12-16

**Authors:** Hoang Le Tuan Anh, Phuong Thao Tran, Do Thi Thao, Duong Thu Trang, Nguyen Hai Dang, Pham Van Cuong, Phan Van Kiem, Chau Van Minh, Jeong-Hyung Lee

**Affiliations:** ^1^Mientrung Institute for Scientific Research, Vietnam Academy of Science and Technology (VAST), 321 Huynh Thuc Khang, Hue city, Thua Thien Hue 531600, Vietnam; ^2^Department of Biochemistry, College of Natural Sciences, Kangwon National University, Chuncheon, Gangwon-Do 200-701, Republic of Korea; ^3^Institute of Biotechnology, VAST, 18 Hoang Quoc Viet, Cau Giay, Hanoi, Vietnam; ^4^Advanced Center for Bio-Organic Chemistry, Institute of Marine Biochemistry, VAST, 18 Hoang Quoc Viet, Cau Giay, Hanoi, Vietnam

## Abstract

Degalactotigonin (**1**) and three other steroidal compounds solasodine (**2**),* O*-acetyl solasodine (**3**), and soladulcoside A (**4**) were isolated from the methanolic extract of* Solanum nigrum*, and their chemical structures were elucidated by spectroscopic analyses. The isolated compounds were evaluated for cytotoxic activity against human pancreatic cancer cell lines (PANC1 and MIA-PaCa2) and lung cancer cell lines (A549, NCI-H1975, and NCI-H1299). Only degalactotigonin (**1**) showed potent cytotoxicity against these cancer cell lines. Compound** 1** induced apoptosis in PANC1 and A549 cells. Further study on its mechanism of action in PANC1 cells demonstrated that** 1** significantly inhibited EGF-induced proliferation and migration in a concentration-dependent manner. Treatment of PANC1 cells with degalactotigonin induced cell cycle arrest at G0/G1 phase. Compound** 1** induced downregulation of cyclin D1 and upregulation of p21 in a time- and concentration-dependent manner and inhibited EGF-induced phosphorylation of EGFR, as well as activation of EGFR downstream signaling molecules such as Akt and ERK.

## 1. Introduction

Pancreatic cancer is the 12th most common cancer worldwide and is the most seventh deathly related-cancer. Surgical resection and chemotherapeutics are the traditional treatment of pancreatic cancer. The recent success of chemotherapy treatments is based on the inhibition of tumor-associated specific pathways and commonly referred to as targeted agents [1]. For instances, some inhibitors of the epidermal growth factor receptors (EGFR) were approved by FDA for the treatment of several cancer types including pancreatic cancer [2–4]. However, these commonly used techniques are frequently challenged in view of metastasis and other pathological changes. Therefore, the search for new antipancreatic cancer agents is important to reduce the mortality rate.

Overexpression of growth factors and their receptors, inactivation of tumor suppressor gene, or activation of oncogene is the stimulators for the growth of an aggressive cancer [5]. EGFR and its family members are the main components of a complex signaling cascade that regulates proliferation, growth, migration, differentiation, and survival of cancer cells [6]. Thirteen known ligands that can activate the EGFR family include epidermal growth factor (EGF), amphiregulin (AR), betacellulin (BTC), transforming growth factor alpha (TGF-*α*), epiregulin (EPR), epigen (EPG), neuregulins 1–6 (NRG), and heparin-binding EGF-like growth factor (HB-EGF) [7]. The activation of EGFR leads to the phosphorylation of specific tyrosine residues on the intracellular cytoplasmic tail, which results in the activation of corresponding signaling cascades critically in cell proliferation, survival, migration, and angiogenesis. EGFR then undergoes endocytosis to recycle or direct to lysosomes for degradation, resulting in the downregulation of EGFR signaling [8]. Receptor tyrosine kinases including EGFR, PDGFR*α* (platelet-derived growth factor receptor-*α*), and VEGFR (vascular endothelial growth factor receptor) have been detected to be activated in pancreatic cancer. Noteworthily, overexpression of EGFR is found up to 90% in pancreatic cancer cells [9]. Though EGFR expression has been supported to growth and metastatic stages of cancer [10], its effects on survival is still a debate [11, 12]. Overall, EGFR is an emerging candidate for further research in pancreatic cancer therapy.

*Solanum nigrum* L. (Solanaceae) has been used in traditional medicine for the treatment of edema, diuretic, inflammation, mastitis, and hepatic cancer [13]. Recent studies showed that the aqueous extract of* S. nigrum* leaves induced autophagy and enhanced cytotoxicity of some chemotherapy drugs in HT-29 human colorectal carcinoma cells [14]. The antitumor properties of extracts from various parts of this plant have been reported [15–18]. Furthermore, the anti-inflammatory, antinociceptive, antineoplastic, antiulcerogenic, and antiviral activities of extracts and compounds of* S. nigrum* were demonstrated [19–25]. Studies on the chemical properties of this plants revealed that alkaloids, glycoproteins, flavonoids, and steroidal glycosides are the major contents, among which, the cytotoxicity of alkaloids and glycoproteins of* S. nigrum* were reported [26–30]. It is assumed that these contents mostly contribute to the antitumor properties of this plant [30–36]. The previous studies indicated that steroidal glycosides from* S. nigrum* are also the major constituents with potential anticancer activities [37–39]. Degalactotigonin, a steroidal glycoside of this plant, showed potent cytotoxicity against multiple cell lines [40]. This compound is considered to be the most cytotoxic steroidal glycoside isolated from* S. nigrum* to date. A recent report demonstrated that this compound suppressed the growth and metastasis of osteosarcoma [41]. In this study, we presented the isolation of some steroidal glycoside from the leaves of* S. nigrum* and evaluated their cytotoxic properties on human lung and pancreatic cell lines. We also investigated for the first time the mechanism of action of cytotoxic degalactotigonin in human pancreatic cancer cell line PANC1.

## 2. Materials and Methods

### 2.1. Plant Materials

The plant* S. nigrum* was collected in August 2015 at Thaibinh province, Vietnam, and was identified by Dr. Do Thanh Tuan, Thaibinh University of Medicine and Pharmacy. The voucher specimen (TB16.2015) was deposited at the Herbarium of Mientrung Institute for Scientific Research (VAST) and Thaibinh University of Medicine and Pharmacy.

### 2.2. Isolation of Compounds** 1**-**4** from* Solanum nigrum*

The whole plant of* S. nigrum* was air dried, ground to powder, and extracted with methanol at 50°C with the aid of ultrasonic (3 times x 1 h each). The organic layer was filtered and removed under vacuum to obtain the crude extract of methanol. This crude extract was suspended in hot distilled water (1.5 L) and successively partitioned with dichloromethane and ethyl acetate (3 times x 1.5 L each) to yield corresponding extracts, dichloromethane (SND, 30 g), ethyl acetate (SNE, 32 g), and water-soluble layer (SNW). The SNW layer was passed through a Diaion HP-20 column, washed with distilled water, and eluted with increasing volume of methanol in water (25%, 50%, 75%, and 100% of methanol) to obtain four subfractions, SNW1–SNW4. The subfraction SNW3 (2,5 g) was chromatographed on a silica gel column and eluted with solvent system of dichloromethane/methanol/water (2.0/1.0/0.1, v/v/v) to obtain four smaller fractions, SNW1A-SNW1D. The fraction SNW1B (0.6 g) was chromatographed on a silica gel column and eluted with dichloromethane/methanol (3.0/1.0, v/v) and then was further purified on an RP-18 reversed phase column and eluted with acetone/water (1.0/2.0, v/v) to yields** 2** (11.0 mg) and** 3** (14.0 mg). The fraction SNW1D (1.2 g) was separated into 2 fractions, SNW2A - SNW2B, on a silica gel column eluting with solvent system dichloromethane/methanol/water (4.0/1.0/0.1, v/v/v). The fraction SNW2A (0.2 g) was further purified on a silica gel column and eluted with dichloromethane/methanol/water (2.0/1.0/0.1, v/v/v) to yield** 4** (7.0 mg). Compound** 1** (50.0 mg) was obtained from fraction SNW2B (0.4 g) on a silica gel column eluting with dichloromethane/acetone/water (1.5/1.0/0.1, v/v/v).

### 2.3. Antibodies and Reagents

EGF was purchased from Invitrogen (Carlsbad, CA, USA). Antibodies including anti-EGFR, anticyclin D1, and anti-p21 were obtained from Santa Cruz Biotechnology (Santa Cruz, CA, USA). Anti-phospho-EGFR, anti-Akt, anti-phospho Akt (Ser473), anti-ERK, and anti-phospho-ERK antibodies were from Cell Signaling Technology (Danvers, MA, USA).

### 2.4. Cell Culture

All cell lines used in this study were obtained from the American Type Culture Collection (Manassas, VA, USA). A549, NCI-H1975, and NCI-H1299 cells were maintained in RPMI 1640 medium. PANC1 and MIA-PaCa2 cells were maintained in DMEM. All media were supplemented with 10% fetal bovine serum (Hyclone, Logan, UT, USA) and streptomycin-penicillin (Invitrogen, Carlsbad, CA, USA). Cultures were maintained in a CO_2_ incubator humidified atmosphere 5% CO_2_ at 37°C.

### 2.5. Cell Viability Assay

The cytotoxic activity of** 1-4 **was determined by MTT [3-(4,5-dimethylthiazol-2-yl)-2,5-diphenyltetrazolium bromide]-based colorimetric assay [42]. MTT was purchased from Sigma, MO, USA. In brief, 2 × 10^5^ cells/mL were seeded into 96-well plate and incubated for overnight. The compounds were treated to each well with various concentrations (0, 1, 3, 10, and 30 *µ*M). After incubation for 48 h, the MTT solution (0.5 mg/mL) was added to each well and further incubated for 3 h. Cell viability was calculated as a percentage compared to the vehicle-only treated control sample and performed in triplicate. The IC_50_ values were calculated using nonlinear regression analysis (percentage survival versus concentration).

### 2.6. Western Blot Analysis

Cells were harvested and lysed in a lysis buffer [150 mM NaCl, 50 mM Tris-HCl, pH 7.4, 1 mM EDTA, and 1% NP-40, 5 mM sodium orthovanadate, and protease inhibitors cocktail (BD Biosciences)] and then centrifuged for 10 min at 4°C and 15,000 rpm. An equal amount of protein was separated onto SDS-PAGE (sodium dodecyl sulfate polyacrylamide gel electrophoresis) and transferred to a Hybond-P membrane (Amersham Biosciences, Buckinghamshire, UK). Membranes were blocked in 5% nonfat skim milk for 1 h at room temperature, probed with the appropriate primary antibodies (1:1,000 dilution), washed, and then incubated with the corresponding secondary antibody (1:2,000 dilution). The signal was detected using the ECL (enhanced chemiluminescence) system (Intron, Seongnam, Korea).

### 2.7. Annexin V and PI Double Staining Assay

Annexin V and PI (propidium iodide) staining for apoptosis detection was performed using an Annexin V–FLOUS kit according to the manufacturer's instructions (BD Biosciences, CA, USA). Briefly, cells were treated with various concentrations of** 1** and incubated for 24 h. The cells were then collected by trypsinization, washed 2 times with cold PBS, suspended in 100 *µ*L of a binding buffer (dilute from 10X binding buffer), and stained with 5 *µ*L PI (stock solution 50 *µ*g/mL) and 5 *µ*L FITC-labeled Annexin V in the dark for 15 min at room temperature. The cells were analyzed by FACS Calibur (Becton Dickinson, CA, USA). The percentages of Annexin V+/PI- (apoptosis cells), Annexin V /PI- (living cells), and Annexin V+ /PI+ (necrotic cells) staining were determined after marking for the positive and negative population.

### 2.8. Cell Cycle Analysis

Cells were seeded into 60 mm culture dishes and serum-starved in DMEM containing 0.5% FBS for 24 h. The cells were treated with indicated concentrations of** 1** for 30 min and then stimulated by EGF (100 ng/mL) for 24 h. The cells were harvested by trypsinization, washed two times with cold PBS, and then centrifuged. The collected cells were fixed in 70% ethanol (v/v) for at least 2 hours at 4°C, washed once with PBS, and then suspended in cold propidium iodide (PI) solution (50 *µ*g/mL) containing RNase A (0.1 *µ*g/mL) in PBS (pH 7.4) in the dark for 15 min at room temperature. Flow cytometry analyses were performed using FACS Calibur (Becton Dickinson, CA, USA). The data were analyzed using the Cell Quest software (Becton Dickinson, CA, USA).

### 2.9. Cell Migration Assay

Cell migration assays were performed by following the Boyden chamber method using polycarbonate membranes with an 8 *μ*m pore size. In brief, 10^5^ cells were seeded onto the upper chamber in 200 *μ*L of DMEM containing 0.5% FBS, and then the upper chamber was put onto the 24-well plate with 800 *μ*L of the same media with or without EGF (100 ng/ml). The various concentrations of** 1** (0, 0.1, 0.3, and 1 *µ*M) were treated in the upper chamber. After 12 h incubation, the migrated cells on the lower surface of the filter were fixed in methanol and then stained with H&E (hematoxylin and eosin). The cotton swabs were used to remove the cells which did not migrate through the pore. The migrated cells were counted in at least five randomly selected microscopic fields (×100) per filter.

### 2.10. Statistical Analysis

Data was expressed as the mean ± standard deviations (SD). Statistical significance was assessed by the two-tailed unpaired Student'*s t-test *and P values less than 0.05 was considered statistically significant.

## 3. Results and Discussion

### 3.1. Isolation and Identification of Compounds** 1**-**4**

**Figure 1:**
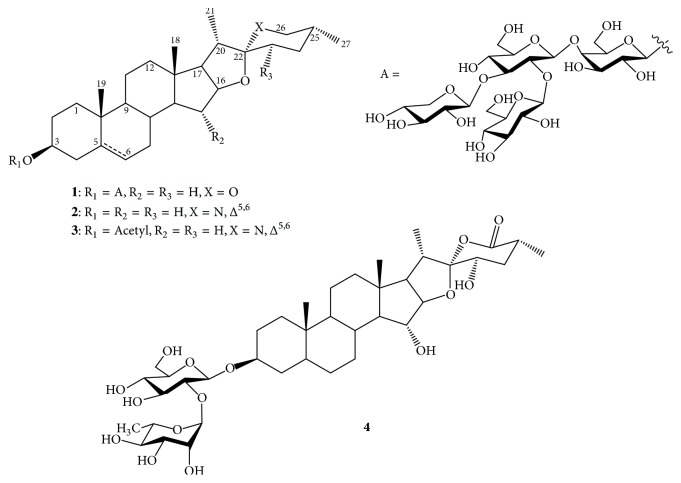
The structures of compounds (**1**-**4**) from* Solanum nigrum.*

Compounds** 1**-**4** were isolated from the methanolic extracts of* S. nigrum* by using various chromatographic techniques. Their structures were elucidated by using spectroscopic data (Supplementary Data (available here)) and by comparison with the reported data [39, 43, 44]. The compounds were determined as degalactotigonin (**1**), solasodine (**2**),* O*-acetyl solasodine (**3)**, and soladulcoside A (**4**) (Figure 1).

### 3.2. Degalactotigonin Induces Apoptosis in PANC1 and A549 Cells

Cytotoxic activities of the isolated compounds on five human cancer cell lines (lung cancer cell lines: A549, NCI-H1975 and NCI-H1299; pancreatic cancer lines: PANC1 and MIA-PaCa2) were evaluated by an MTT method. As a result, compound** 1** showed to be the most active compound against all tested cell lines (Table 1). The results were in accordance with those of previous reports in which degalactotigonin showed the cytotoxicity against various cancer cell lines including PC-12 (human lung carcinoma), HCT-116 (human colon carcinoma), NCI-H460 (human lung carcinoma), HepG2 (human liver carcinoma), MCF-7 (human breast carcinoma), and SF-268 (human glioma) [39, 40]. Further studies demonstrated that this compound induced apoptosis in MCF-7 cells and suppressed the growth and metastasis of human osteosarcoma through modulation of GSK-3*β* inactivation–mediated repression of the Hedgehog/Gli1 pathway [41, 45].

**Figure 2:**
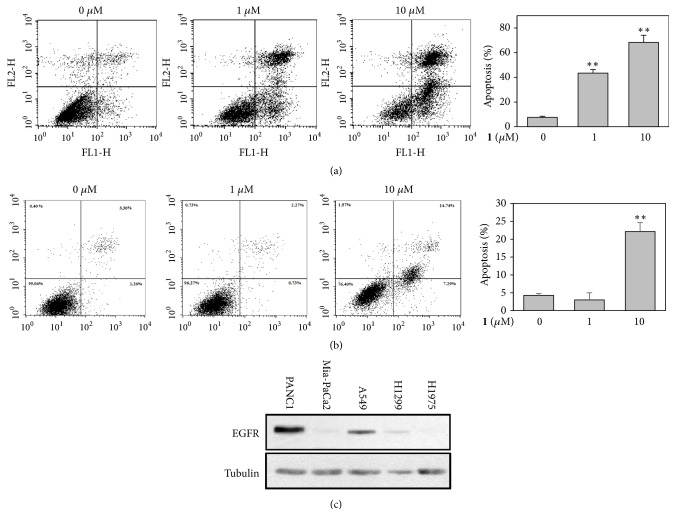
Effect of** 1** on apoptosis of PANC1 and A549 cells. (a) PANC1 or (b) A549 cells were treated with the indicated concentrations of 1 (0-10 *μ*M) for 12 h, and subsequently stained with Annexin V-FITC and PI. The percentage of Annexin V-FITC positive apoptotic cells was analyzed by flow cytometry. The results were presented in three independent experiments and described as the mean ± SD. *∗*, P<0.05; *∗∗*, P<0.01 versus vehicle-treated control. (c) Expression of EGFR in PANC1, MIA-PaCa2, A549, NCI-H1975, and NCI-H1299 cell lines. Whole cell lysates from these cell lines were probed with the indicated antibodies. *α*-Tubulin was used as a loading control.

We determined apoptosis-inducing activity of** 1** in PANC1 and A549 cells by Annexin V/PI double staining assays. Treatment with** 1** increased the ration of apoptotic cells (Annexin V positive) in a concentration-dependent manner in these cells and PANC1 cells were more sensitive to apoptosis induced by** 1** than A549 cells (Figures 2(a) and 2(b)). Since PANC1 cells were the most sensitive to** 1** (Table 1) and expressed the highest level of EGFR compared to other tested cell lines (Figure 2(c)), we further investigated on the mechanism of action of** 1** in PANC1 cells.

### 3.3. Degalactotigonin Inhibits EGF-Induced Cell Cycle Progression of PANC1 Cells

**Figure 3:**
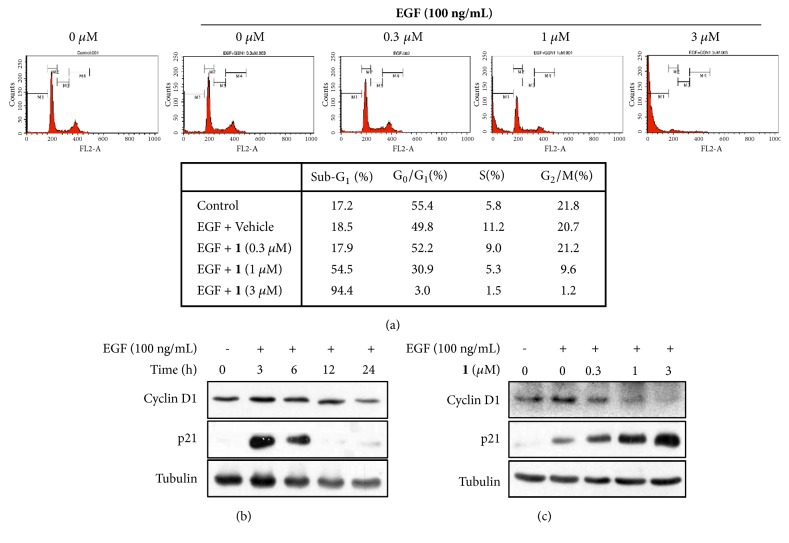
Effect of** 1** on EGF-induced cell cycle progression of PANC1 cells. (a) Serum-starved PANC1 cells were pretreated with the indicated concentrations of** 1** (0-3 *μ*M) for 30 min and then stimulated with EGF (100 ng/mL) for 24 h, stained with propidium iodide, and analyzed using a flow cytometry. Histogram showed the percentage of cells within the sub-G1, G0/G1, S, and G2/M phases of the cell cycle. Data was expressed as means of at least three independent experiments. (b) Serum-starved PANC1 were incubated with EGF (100 ng/mL) for the indicated time and whole cell lysates were probed with the indicated antibodies. *α*-Tubulin was used as a loading control. (c) Serum-starved PANC1 cells were incubated with EGF (100 ng/mL) in the presence of the indicated concentrations of** 1** for 3 hours and whole cell lysates were probed with the indicated antibodies. *α*-Tubulin was used as a loading control.

EGF acts as a regulator of cell proliferation, growth and migration by binding to its receptor EGFR [46]. We determined whether** 1** inhibits EGF-induced cell cycle progression of PANC1 cells. Serum starvation of PANC1 cells for 24 h resulted in approximately 55.% synchronization of the cell cycle at the G_0_/G_1_ phase. The percentage of S phase cells increased from 5.8% to 11.2% after treatment with EGF (100 ng/mL) for 24 h. In contrast, treatment with** 1** at 0.3 *μ*M significantly induced cell cycle arrest at G_0_/G_1_ phase by increasing the percentage of cells up to 52.2% and reducing the S phase to 9.0% (Figure 3(a)), suggesting that** 1** could inhibit EGF-induced cell cycle progression via inhibiting EGFR signaling pathway. However, treatment with higher concentration of** 1** (1 *μ*M and 3 *μ*M) induced cell death due to its cytotoxicity in serum-starved condition.

Cyclin D1 and p21 are two important regulators in the G_1_/S phase progression and downstream target genes of the EGFR signaling pathway. p21 (also known as Waf1/Cip1), a well-known cyclin-dependent kinase inhibitor, is involved in regulation of the cell cycle and acts as a mediator of cell survival as well as inhibiting DNA synthesis [47, 48]. Overexpression of p21 can potentially inhibit cyclin D1/CDK4 complex and suppress the catalytic activity of this complex, leading to cell cycle arrest at G1 phase [49]. Western blot analyses revealed that stimulation of PANC1 cells with EGF increased the expression levels of cyclin D1 and p21 in a time-dependent manner (Figure 3(b)). Treatment of PANC1 cells with** 1** inhibited EGF-induced expression of cyclin D1 (Figure 3(c)), suggesting that** 1** could inhibit EGFR signaling pathway. However, treatment with** 1** further increased the expression level of p21 in PANC1 cells (Figure 3(c)). In osteosarcoma cancer cells,** 1** increases p21 mRNA and protein expression through undefined mechanisms and induces cell cycle arrest [41]. Thus, it is likely that** 1** induces p21 expression via EGFR-independent signaling pathway and the induction of p21 by** 1** may significantly contribute to the inhibition of EGF-induced cell cycle progression.

### 3.4. Degalactotigonin Suppresses EGF-Induced Activation of EGFR in PANC1 Cells

**Figure 4:**
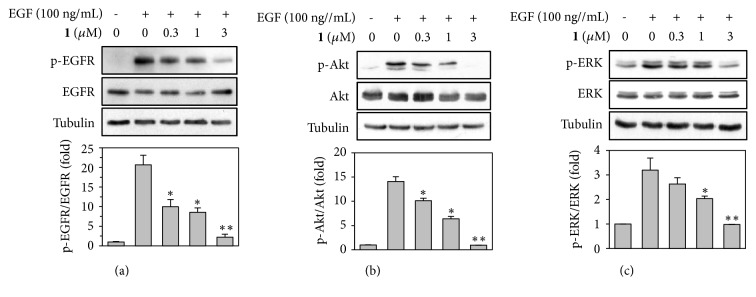
Effect of for** 1** on EGF-induced activation of EGFR in PANC1 cells. (a, b, and c) Serum-starved PANC1 cells were stimulated with EGF (100 ng/mL) in the presence of the indicted concentrations of** 1** for 5 min. Whole cell lysates were prepared and Western blotting was performed to determine the expression level of p-EGFR (a), EGFR (a), p-Akt (b), Akt (b), p-ERK (c), and ERK (c). *α*-Tubulin was used as a loading control. The blots were quantified by Image J software and the levels of p-EGFR, p-Akt, and p-ERK (normalized to EGFR, Akt, and ERK, respectively) were expressed as the mean ± SD of three independent experiments. *∗*P<0.05, *∗∗*P<0.01 versus EGF only treated control.

After EGF binding to EGFR in the cell membrane, EGFR is autophosphorylated, endocytosis, and recycled or directed to lysosomes for degradation [50]. We determined whether** 1** regulates EGFR activation in response to EGF stimulation. Serum-starved PANC1 cells were treated with** 1** at different concentrations for 30 min followed by EGF stimulation for 5 min. Treatment with** 1** significantly reduced the level of EGFR phosphorylation (Y1068) (Figure 4(a)), suggesting that** 1** may inhibit EGFR activation. Next, we examined whether** 1** inhibits the activation of EGFR downstream signaling molecules such as Akt and ERK (extracellular signal-regulated kinase) [33]. The PI3K (phosphatidylinositol 3-kinase)/Akt pathway is activated in many cancers, and inhibition of the PI3K/Akt pathway induces cell apoptosis [51]. The activation of Ras/Raf/ERK by growth factor triggers the synthesis of D-type cyclins which bind with Cdk4 or Cdk6 to regulate cell cycle progression [52]. Treatment of PANC1 cells with** 1** inhibited EGF-induced Akt (Ser473) and ERK (Thr202/Tyr204) phosphorylation (Figures 4(b) and 4(c)) in a concentration-dependent manner. Taken together, these results suggested that** 1** may inhibit EGF-mediated activation of EGFR and subsequent EGFR downstream signaling molecules such as Akt and ERK.

### 3.5. Degalactotigonin Attenuates EGF-Induced Migration of PANC1 Cells

**Figure 5:**
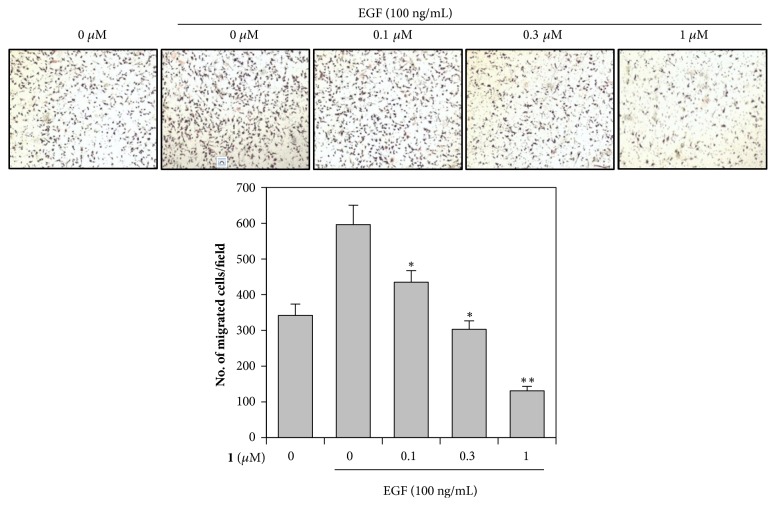
Effect of** 1** on EGF-induced migration of PANC1 cells. PANC1 cells were treated with the indicated concentrations of** 1** and then underwent a transwell invasion assay in the absence or presence of EGF (100 ng/mL) for 12 h. Representative images were shown (top). The graphs represented quantification of migrated PANC1 cells (bottom) and the mean ± SD of three independent experiments was expressed. *∗*P<0.05, *∗∗*P<0.01 versus EGF only treated control.

In the tumor progression, acquisition of the invasive and metastatic capability is important characteristics, which correlate with poor clinical prognosis and become the barrier to successful treatment [53]. EGF is a well-known growth factor that promotes cancer cell migration and motility [54–56]. To further investigate the inhibitory effect of** 1** on EGF-induced signaling, we determined whether** 1** inhibits EGF-induced migration of PANC1 cells by transwell migration assay. The results showed that** 1** markedly decreased the migration of PANC1 cells induced by EGF in a dose-dependent manner (Figure 5), suggesting that** 1** effectively suppressed EGF-mediated migration of PANC1 cells via inhibiting EGFR signaling pathway.

Pancreatic cancer is one of the most common deathly cancers in the Western world [57]. Overexpression of EGFR has been detected up to 90% in this cancer [9]. Gefitinib and erlotinib were the first EGFR tyrosine kinase inhibitors to be developed. Both of them target blocking EGFR signaling downstream pathways as competitive inhibitors of ATP for the tyrosine kinase domain [58]. Degalactotigonin (**1**), a saponin isolated from* Solanum nigrum*, has chemopreventive effects on various cancer types [45]. However, the anticancer effects of** 1** and its mode of action mechanism in pancreatic cancer cells have not been investigated yet. In this study, we demonstrated that** 1** induced apoptosis in PANC cells. Notably, compound** 1** reduced EGF-induced phosphorylation of EGFR and its downstream signaling molecules such as Akt and ERK and suppressed the EGF-induced proliferation and migration of PANC1 cells in a dose-dependent manner.

Taken together, this study showed that** 1** not only suppressed proliferation and migration of PANC1 cells, but also induced cell cycles arrest at G0/G1 phase and apoptosis via inhibiting EGF-mediated EGFR activation. These results might clarify a novel biological mechanism for** 1** in antitumor therapy targeted on EGFR-overexpressing pancreatic cancer.

## Figures and Tables

**Table 1:** Cytotoxic activities of steroidal glycosides from *S. nigrum* on five cancer cell lines.

Compounds	IC_50_ (*µ*M)^a^				
	A549	H1975	H1299	PANC1	MIA-PaCa2
**1**	4.9 ± 1.0	5.5 ± 0.6	6.3 ± 0.8	2.9 ± 0.2	6.4 ± 0.4
**2**	>30	>30	>30	>30	>30
**3**	>30	>30	27.9 ± 3.0	>30	>30
**4**	28.4 ± 3.1	>30	>30	>30	>30
Camptothecin^b^	1.5 ± 0.9	0.16 ± 0.1	0.24 ± 0.1	4.7 ± 0.5	3.3 ± 0.2

^a^The IC_50_ values were calculated in a triplicate experiment.

^b^Camptothecin used as positive control.

## Data Availability

The data used to support the findings of this study are included within the supplementary information file.

## Supplementary Materials

Supplementary Materials The spectroscopic data of the isolated compounds used to support the findings of this study are included within the supplementary information file.
