# Supplementary material for: Degalactotigonin, a Steroidal Glycoside from Solanum nigrum, Induces Apoptosis and Cell Cycle Arrest via Inhibiting the EGFR Signaling Pathways in Pancreatic Cancer Cells
Source: Biomed Res Int. 2018 Dec 16;2018:3120972. doi: 10.1155/2018/3120972 (PMC6311251; doi:10.1155/2018/3120972)
Supplement: Supplementary Materials — The spectroscopic data of the isolated compounds used to support the findings of this study are included within the supplementary information file. [file 3120972.f1.docx]

**Supplement Material for**

Degalactotigonin, a steroidal glycoside from Solanum nigrum, induces apoptosis and cell cycle arrest via inhibiting the EGFR signaling pathways in pancreatic cancer cells

Hoang Le Tuan Anh^a,†^, Tran Phuong Thao^b,†^, Do Thi Thao^c^, Duong Thu Trang^d^, Nguyen Hai Dang^d,^*, Pham Van Cuong^d^, Phan Van Kiem^d^, Chau Van Minh^d^, Jeong-Hyung Lee^b,^*

*^a^Mientrung Institute for Scientific Research, Vietnam Academy of Science and Technology (VAST), 321 Huynh Thuc Khang, Hue city, Thua Thien Hue 531600, Vietnam*

*^b^Department of Biochemistry, College of Natural Sciences, Kangwon National University, Chuncheon, Gangwon-Do 200-701, Republic of Korea*

*^c^Institute of Biotechnology, VAST, 18 Hoang Quoc Viet, Cau Giay, Hanoi, Vietnam*

*^d^Advanced Center for Bio-organic Chemistry, Institute of Marine Biochemistry, VAST, 18 Hoang Quoc Viet, Cau Giay, Hanoi, Vietnam*

Physiochemical data of compounds isolated from *Solanum nigrum*:

**Compound 1: Desgalactotigonin**

- White amorphous powder;

- Molecular formular: C_62_H_100_O_26_;

- Molecular weight: 1261

^1^H-NMR (DMSO-*d_6_*, 500 MHz): δ_H_ 0.91 (1H, m, Ha-1), 1.67 (1H, m, Hb-1), 1.29 (1H, m, Ha-2), 1.57 (1H, m, Hb-2), 3.14 (1H, m, H-3), 1.17 (1H, m, Ha-4), 1.65 (1H, m, Hb-4), 1.01 (1H, m, H-5), 1.23 (1H, m, Ha-6), 1.59 (1H, m, Hb-6), 0.88 (1H, m, Ha-7), 1.89 (1H, m, Hb-7), 1.49 (1H, m, H-8), 0.63 (1H, br t, *J* = 9.0 Hz, H-9), 1.20 (1H, m, Ha-11), 1.45 (1H, m, Hb-11), 1.10 (1H, m, Ha-12), 1.55 (1H, m, Hb-12), 1.04 (1H, m, H-14), 1.10 (1H, m, Ha-15), 1.89 (1H, m, Hb-15), 4.26 (1H, dd, *J* = 7.5, 14.5 Hz, H-16), 1.65 (1H, m, H-17), 0.71 (3H, s, CH_3_-18), 0.77 (3H, s, CH_3_-19), 1.80 (1H, t, *J* = 7.0 Hz, H-20), 0.89 (1H, d, *J* = 7.0 Hz, CH_3_-21), 1.49 (1H, m, Ha-23), 1.61 (1H, m, Hb-23), 1.39 (1H, m, Ha-24), 1.72 (1H, m, Hb-24), 1.51 (1H, m, H-25), 3.40 (1H, overlapped, Ha-26), 3.23 (1H, dd, *J* = 5.0, 10.5 Hz, Hb-26), 0.73 (3H, d, *J* = 6.5 Hz, CH_3_-27), 4.20 (1H, d, *J* = 7.5 Hz, H-1′), 3.05 (1H, overlapped, H-2′), 3.22(1H, overlapped, H-3′), 3.77(1H, overlapped, H-4′), 3.52 (1H, overlapped, H-5′), 3.39 (1H, overlapped, Ha-6′), 3.74 (1H, overlapped, Hb-6′), 4.41 (1H, d, *J* = 7.5 Hz, H-1″), 3.58 (1H, overlapped, H-2″), 3.60 (1H, overlapped, H-3″), 3.14 (1H, overlapped, H-4″), 3.15 (1H, overlapped, H-5″), 3.38 (1H, overlapped, Ha-6″), 3.75 (1H, overlapped, Hb-6″), 4.32 (1H, d, *J* = 8.0 Hz, H-1″′), 2.96 (1H, overlapped, H-2″′), 3.15 (1H, overlapped, H-3″′), 3.32 (1H, overlapped, H-4″′), 3.53 (1H, overlapped, H-5″′), 3.90 (1H, dd, *J* = 2.5, 11.5 Hz, Ha-6″′), 3.59 (1H, dd, *J* = 5.5, 11.5 Hz, Hb-6″′), 4.49 (1H, d, *J* = 8.0 Hz, H-1″″), 3.51 (1H, overlapped, H-2″″), 3.14 (1H, overlapped, H-3″″), 3.30 (1H, overlapped, H-4″″), 3.11 (1H, dd, *J* = 4.5, 11.0 Hz, Ha-5″″) and 3.77 (1H, dd, *J* = 2.0, 11.0 Hz, Hb-5″″).

^13^C-NMR (DMSO-*d_6_*, 125MHz): δ_C_ 36.5 (C-1), 28.5 (C-2), 76.4 (C-3), 34.0 (C-4), 44.0 (C-5), 28.3 (C-6), 31.8 (C-7), 34.6 (C-8), 53.7 (C-9), 35.3(C-10), 20.6 (C-11), 38.9 (C-12), 40.0 (C-13), 55.6 (C-14), 31.4 (C-15), 80.2 (C-16), 61.9 (C-17), 16.2 (C-18), 12.1 (C-19), 41.1 (C-20), 14.6 (C-21), 108.4 (C-22), 30.9 (C-23), 29.0 (C-24), 29.8 (C-25), 65.9 (C-6), 17.1 (C-7), 100.9 (C-1′), 73.5 (C-2′), 75.9 (C-3′), 78.9 (C-4′), 76.1(C-5′), 59.5 (C-6′), 103.3 (C-1″), 79.3 (C-2″), 85.0 (C-3″), 69.7 (C-4″), 76.5 (C-5″), 61.3 (C-6″), 102.4 (C-1″′), 74.3 (C-2″′), 76.9 (C-3″′), 69.4 (C-4″′), 76.5 (C-5″′), 60.9 (C-6″′), 103.3 (C-1″″), 73.8 (C-2″″), 76.1 (C-3″″), 68.8 (C-4″″) and 65.9 (C-5″″).

**Compound 2: Solasodine**

- white amorphous powder

- molecular formula: C_27_H_43_NO_2_

- molecular weight: 413

^1^H-NMR (pyridine-*d_5_*, 500 MHz): δ_H_ 0.88 (1H, m, Ha-1), 1.59 (1H, m, Hb-1), 2.02 (2H, m, H-2), 3.71 (1H, m, H-3), 2.51 (2H, m, H-4), 5.23 (1H, br s, H-6), 1.65 (1H, m, Ha-7), 1.99 (1H, m, Hb-7), 1.36 (1H, m, H-8), 0.76 (1H, m, H-9), 1.21 (2H, m, H-11), 0.98 (1H, m, Ha-12), 1.54 (1H, m, Hb-12), 0.94 (1H, m, H-14), 1.30 (1H, m, Ha-15), 1.64 (1H, m, Hb-15), 4.46 (1H, br d, *J* = 6.0, H-16), 1.80 (1H, br d, *J* = 7.0 Hz, H-17), 0.76 (3H, s, CH_3_-18), 0.88 (3H, s, CH_3_-19), 1.90 (1H, t, *J* = 5.5 Hz, H-20), 1.12 (1H, d, *J* = 5.5 Hz, CH_3_-21), 1.63 (1H, m, Ha-23), 1.78 (1H, m, Hb-23), 1.54 (2H, m, H-24), 1.69 (1H, m, H-25), 2.68 (1H, t, *J* = 5.5 Hz, Ha-26), 2.66 (1H, br d, *J* = 5.5 Hz, Hb-26) and 0.70 (3H, d, *J* = 6.0 Hz, CH_3_-27).

^13^C-NMR (pyridine-*d_5_*, 125 MHz): δ_C_ 37.0 (C-1), 31.1 (C-2), 70.8 (C-3), 41.8 (C-4), 141.1 (C-5), 120.7 (C-6), 31.6 (C-7), 31.0 (C-8), 49.7 (C-9), 36.3 (C-10), 20.5 (C-11), 39.3 (C-12), 40.0 (C-13), 56.9 (C-14), 31.7 (C-15), 78.8 (C-16), 62.2 (C-17), 15.9 (C-18), 18.9 (C-19), 41.3 (C-20), 15.0 (C-21), 98.3 (C-22), 33.5 (C-23), 30.0 (C-24), 30.1 (C-25), 46.8 (C-66) and 19.1 (C-27).

**Compound 3: O-acetylsolasodine**

- white amorphous powder

- molecular formula: C_29_H_45_NO_3_

- weight formula: 455

^1^H-NMR (CDCl_3_, 500 MHz): δ_H_ 1.16 (1H, m, Ha-1), 1.85 (1H, m, Hb-1), 1.57 (1H, m, Ha-2), 1.87 (1H, m, Hb-2), 4.59 (1H, m, H-3), 2.33 (1H, br d, *J* = 5.0 Hz, H-4), 5.37 (1H, br d, *J* = 5.0Hz, H-6), 2.14 (2H, m, H-7), 1.68 (1H, m, H-8), 0.72 (1H, m, H-9), 1.53 (2H, m, H-11), 1.19 (1H, dt, *J* = 3.5, 12.5 Hz, Ha-12), 1.68 (1H, dd, *J* = 5.0, 12.5 Hz, Hb-12), 1.71 (1H, t, *J* = 8.0 Hz, H-14), 2.20 (1H, m, H-15), 4.28 (1H, q, *J* = 7.0, 15.0 Hz, H-16), 1.70 (1H, overlapped, H-17), 0.82 (3H, s, CH_3_-18), 1.04 (3H, s, CH_3_-19), 1.90 (1H, dd, *J* = 7.0, 14.0 Hz, H-20), 0.94 (1H, d, *J* = 7.0 Hz, CH_3_-21), 1.59 (1H, m, Ha-23), 1.63 (1H, m, Hb-23), 1.38 (1H, m, Ha-24), 1.60 (1H, m, Hb-24), 1.54 (1H, m, H-25), 2.60 (1H, t, J = 11.0 Hz, Ha-26), 2.66 (1H, dd, J = 3.5, 11.0), 0.84 (3H, d, *J* = 7.5 Hz, CH_3_-27) and 2.03 (3H, s, H-2′).

^13^C-NMR (CDCl_3_, 125 MHz): δ_C_ 37.0 (C-1), 27.7 (C-2), 73.9 (C-3), 38.1 (C-4), 139.7 (C-5), 122.3 (C-6), 32.1 (C-7), 31.4 (C-8), 50.0 (C-9), 36.7 (C-10), 20.8 (C-11), 39.9 (C-12), 40.5 (C-13), 56.4 (C-14), 32.2 (C-15), 78.7 (C-16), 62.8 (C-17), 16.4 (C-18), 19.3 (C-19), 41.2 (C-20), 15.3 (C-21), 98.3 (C-22), 34.1 (C-23), 30.3 (C-24), 31.4 (C-25), 47.7 (C-66), 19.3 (C-27), 170.6 (C-1′) and 21.4 (C-2′).

**Compoud 4: Soladucoside A**

- white amorphous powder

- molecular formula: C_39_H_62_O_15_

- molecular weight: 770

^1^H-NMR (MeOD, 500 MHz): δ_H_ 1.00 (1H, m, Ha-1), 1.74 (1H, m, Hb-1), 1.33 (1H, m, Ha-2), 1.90 (1H, m, Hb-2), 3.60 (1H, m, H-3), 1.37 (1H, m, Ha-4), 1.72 (1H, m, Hb-4), 1.11 (1H, t, *J* = 7.5 Hz, H-5), 1.30 (1H, m, Ha-6), 1.87 (1H, m, Hb-6), 1.08 (1H, m, Ha-7), 2.13 (1H, dd, *J* = 4.0, 13.5 Hz, Hb-7), 1.78 (1H, dd, *J* = 3.5, 11.0 Hz, H-8), 0.72 (1H, dt, *J* = 4.0, 12.0 Hz, H-9), 1.35 (1H, m, Ha-11), 1.56 (1H, m, Hb-11), 1.21 (1H, m, Ha-12), 1.68 (1H, m, Hb-12), 1.21 (1H, m, H-14), 3.80 (1H, dd, *J* = 4.0, 11.0 Hz, H-15), 4.35 (1H, dd, *J* = 4.5, 9.5 Hz, H-16), 1.93 (1H, m, H-17), 0.83 (3H, s, CH_3_-18), 0.88 (3H, s, CH_3_-19), 2.47 (1H, t, *J* = 7.0 Hz, H-20), 1.03 (1H, d, *J* = 7.0 Hz, CH_3_-21), 4.40 (1H, dd, *J* = 2.0, 8.5 Hz, H-23), 1.94 (1H, dd, *J* = 9.0, 13.0 Hz, Ha-24), 2.74 (1H, ddd, *J* = 2.0, 9.0, 12.5 Hz, Hb-24), 2.91 (1H, m, H-25), 1.22 (3H, d, *J* = 7.5 Hz, CH_3_-27), 4.46 (1H, d, *J* = 7.5 Hz, H-1′), 3.63 (1H, t, *J* = 8.0 Hz, H-2′), 3.50 (1H, t, *J* = 6.0 Hz, H-3′), 3.81 (1H, overlapped, H-4′), 3.74 (1H, overlapped, H-5′), 3.73 (2H, overlapped, H-6′), 5.17 (1H, d, *J* = 1.5 Hz, H-1″), 3.93 (1H, dd, *J* = 2.0, 9.0 Hz, H-2″), 3.68 (1H, *J* = 3.5, 9.0 Hz,, H-3″), 3.41 (1H, t, *J* = 9.0 Hz, H-4″), 4.14 (1H, *J* = 6.0, 9.0 Hz,, H-5″) and 1.26 (3H, d, *J* = 7.5, Hb-6″).

^13^C-NMR (MeOD, 125 MHz): δ_C_ 38.4 (C-1), 30.0 (C-2), 76.2 (C-3), 35.1 (C-4), 46.0 (C-5), 29.8 (C-6), 33.3 (C-7), 37.0 (C-8), 55.8 (C-9), 36.8 (C-10), 22.0 (C-11), 41.8 (C-12), 42.3 (C-13), 61.3 (C-14), 80.0 (C-15), 92.0 (C-16), 60.6 (C-17), 18.1 (C-18), 12.9 (C-19), 37.9 (C-20), 15.2 (C-21), 110.4 (C-22), 78.8 (C-23), 31.5 (C-24), 35.1 (C-25), 183.3 (C-66), 16.3 (C-27), 100.7 (C-1′), 77.2 (C-2′), 76.3 (C-3′), 70.9 (C-4′), 78.5 (C-5′), 62.4 (C-6′), 102.2 (C-1″), 72.2 (C-2″), 72.4 (C-3″), 73.9 (C-4″), 69.7 (C-5″) and 18.0 (C-6″).
